# Supplementary figures and images for: Impact of time and temperature on gut microbiota and SCFA composition in stool samples
Source: PLoS One. 2020 Aug 3;15(8):e0236944. doi: 10.1371/journal.pone.0236944 (PMC7398539; doi:10.1371/journal.pone.0236944)

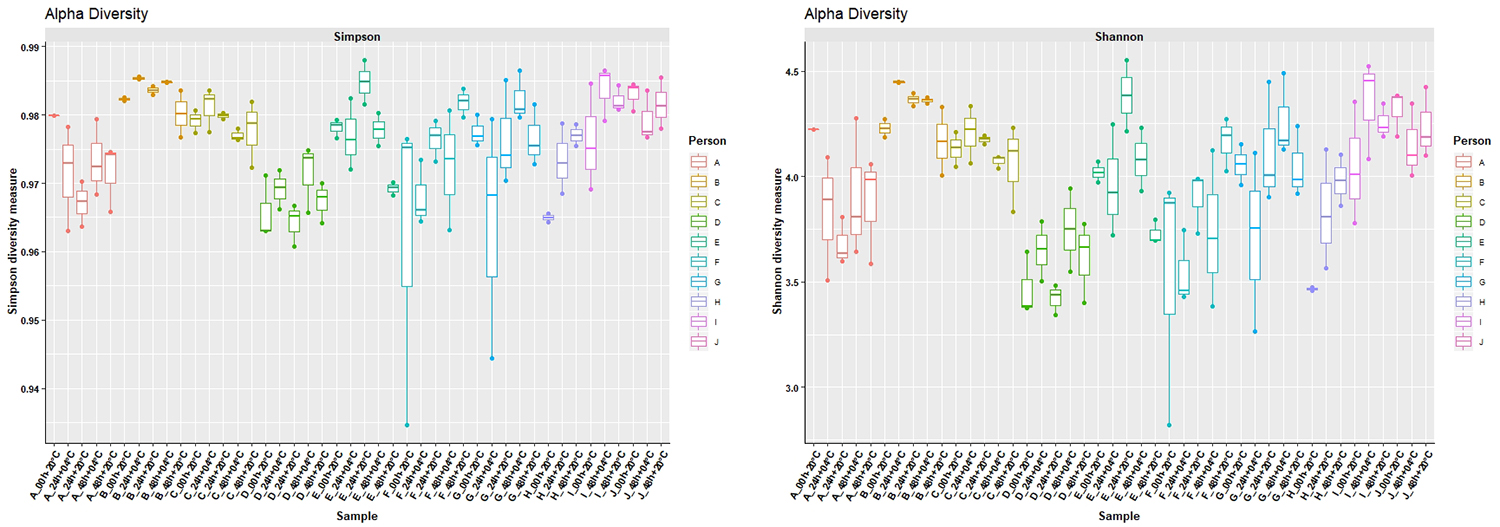

Supplement: S1 Fig — Individuals are indicated by different colors and capitals A-J. Extended lines indicate variability outside the upper and lower limits of the box, whereas outliers are plotted as individual points. (JPEG) [file pone.0236944.s001.jpeg]

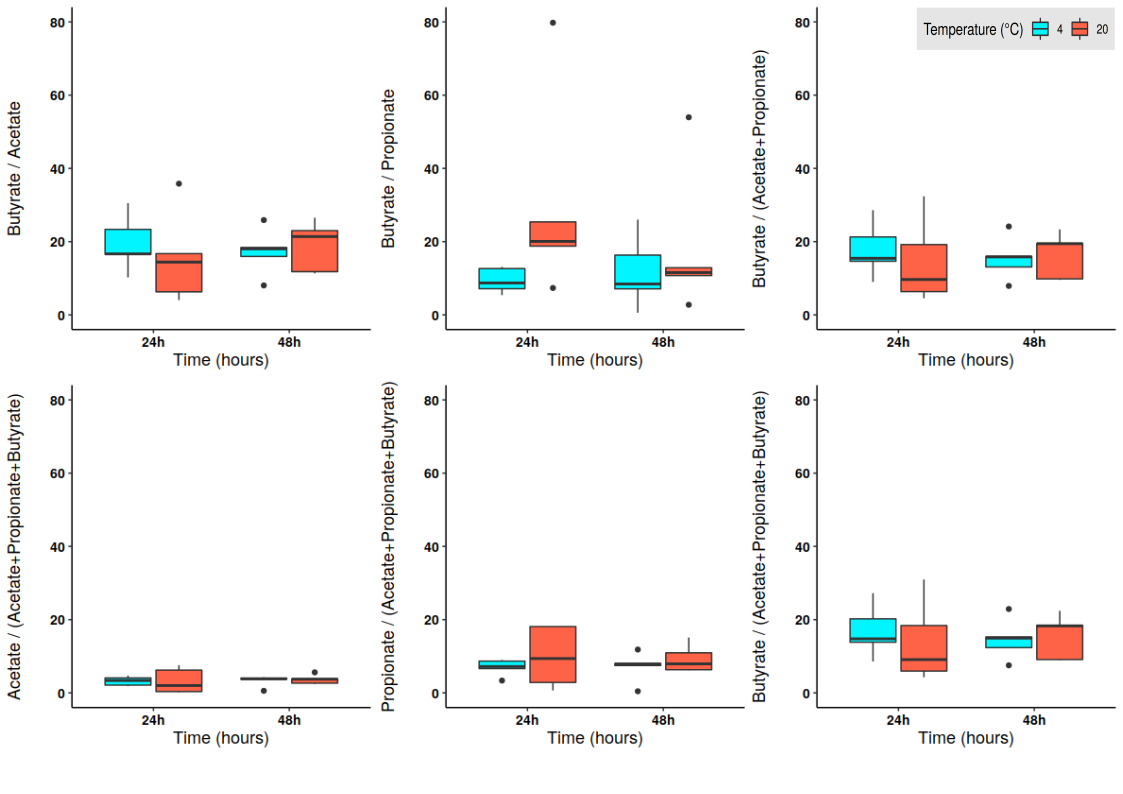

Supplement: S3 Fig — Blue: samples stored at 4°C, red: samples stored at 20°C. Extended lines indicate variability outside the upper and lower quantity of the box, whereas outliers are plotted as individual points. (TIFF) [file pone.0236944.s003.tiff]
