# Supplementary material for: Impact of time and temperature on gut microbiota and SCFA composition in stool samples
Source: PLoS One. 2020 Aug 3;15(8):e0236944. doi: 10.1371/journal.pone.0236944 (PMC7398539; doi:10.1371/journal.pone.0236944)

Figure S2: Effect of temperature and time on SCFA concentration and SCFA ratios displayed as deviation in % from the initial values obtained from immediately frozen samples.

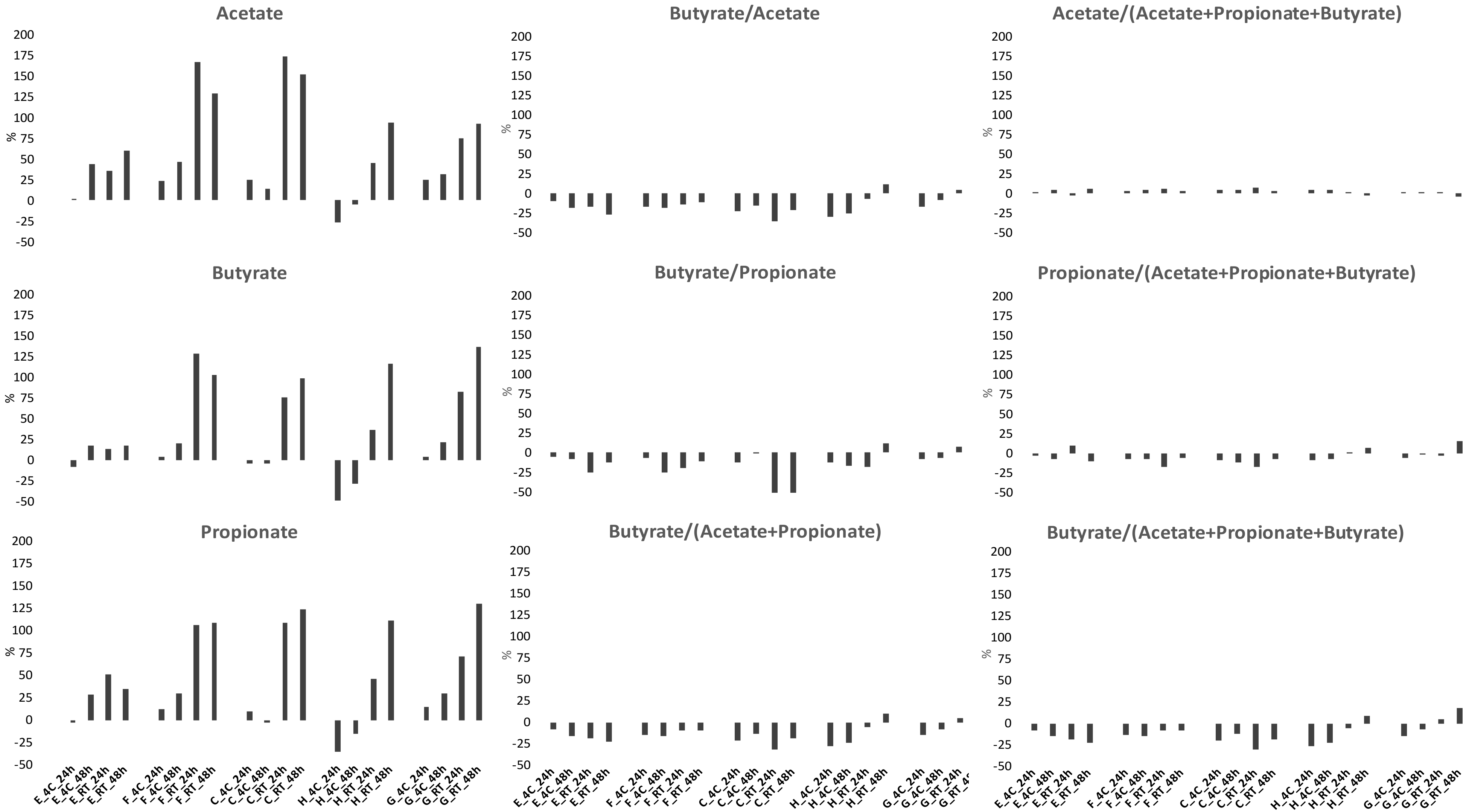

Supplement: S2 Fig — (PDF) [file pone.0236944.s002.pdf]
